# Strategies for Enhancing the Sensitivity of Electrochemiluminescence Biosensors

**DOI:** 10.3390/bios12090750

**Published:** 2022-09-11

**Authors:** Yueyue Huang, Yuanyuan Yao, Yueliang Wang, Lifen Chen, Yanbo Zeng, Lei Li, Longhua Guo

**Affiliations:** 1 Jiaxing Key Laboratory of Molecular Recognition and Sensing, College of Biological, Chemical Sciences and Engineering, Jiaxing University, Jiaxing 314001, China; 2 College of Chemistry and Life Sciences, Zhejiang Normal University, Jinhua 321004, China

**Keywords:** DNA, biosensor, electrochemiluminescence, sensitivity, amplification strategy

## Abstract

Electrochemiluminescence (ECL) has received considerable attention as a powerful analytical technique for the sensitive and accurate detection of biological analytes owing to its high sensitivity and selectivity and wide dynamic range. To satisfy the growing demand for ultrasensitive analysis techniques with high efficiency and accuracy in complex real sample matrices, considerable efforts have been dedicated to developing ECL strategies to improve the sensitivity of bioanalysis. As one of the most effective approaches, diverse signal amplification strategies have been integrated with ECL biosensors to achieve desirable analytical performance. This review summarizes the recent advances in ECL biosensing based on various signal amplification strategies, including DNA-assisted amplification strategies, efficient ECL luminophores, surface-enhanced electrochemiluminescence, and ratiometric strategies. Sensitivity-enhancing strategies and bio-related applications are discussed in detail. Moreover, the future trends and challenges of ECL biosensors are discussed.

## 1. Introduction

Electrogenerated chemiluminescence (ECL) is the phenomenon resulting from electrogenerated species undergoing an electron-transfer reaction at the electrode surface, resulting in the emission of light [1,2,3]. ECL has received a large amount of attention due to its advantages of high sensitivity, low background noise, spatial and temporal control, and no required light source [4,5,6,7]. The first detailed ECL studies were reported in the 1960s by Hercules and Bard [8,9]. Since then, ECL has gradually become a major area of research, with studies encompassing fundamental studies, reagent development, and analytical applications. Many reviews on the details and on our comprehensive understanding of ECL have been published [10,11,12,13]. So far, ECL has been widely applied in various fields, including in food safety, environmental monitoring, and medical diagnosis.

In recent years, ECL biosensors have gradually attracted increasing interest in the field of bioanalysis. Critically, they show great promise for clinical diagnostics and pharmaceutical analysis. Their significant advantages of portability, high sensitivity, and simple operation promote their further development. Moreover, biosensors can provide fast responses at low costs [14,15,16]. Despite their many merits, the development of biosensors for use in the sensitive and accurate detection of analytes at trace levels with high efficiency and accuracy in complex conditions has represented a critical need in many areas. Specifically, the precision and sensitive measurement of protein biomarkers has great significance and practical value in early diagnosis for disease prediction.

**Scheme 1:**
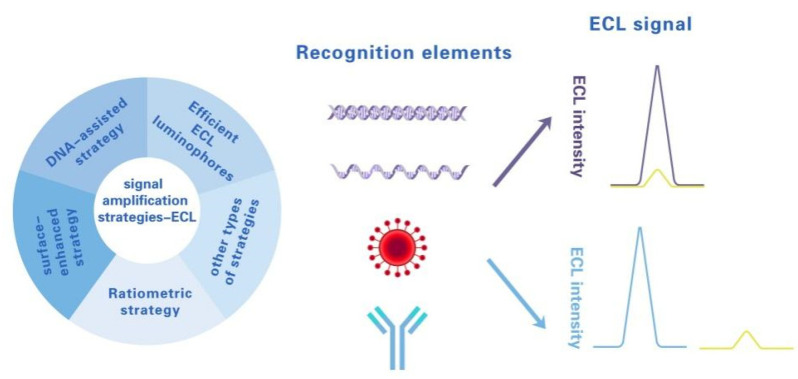
Overview of signal amplification strategies integrated with ECL biosensors for single-signal or multiple-signal outputs.

As shown in Scheme 1, biosensing is a process that converts biochemical interactions into output signals for the quantitative determination of target molecules. Double-stranded DNA, single-stranded DNA, antigens, and antibodies are normally employed as recognition elements for biosensor construction. The main signal output modes include single-signal output and multiple-output. Signal amplification is often considered to be one of the most effective strategies for efficient signal transduction and to amplify signal output [17]. Signal amplification-based biosensors ideally possess the features of enhanced sensitivity and selectivity and a wide dynamic range compared to conventional biosensors [18]. Currently, successful signal amplification strategies that are performed in the ECL realm mainly focus on DNA-assisted amplification strategies, improving the efficiency of ECL luminophores and surface-enhanced electrochemiluminescence, ratiometric strategies, and so on. In this review, we have summarized the recently developed and main ECL bioanalysis strategies, with a more detailed emphasis on advanced DNA signal amplification technologies. Finally, the future trends and perspectives of strategies in ECL bioanalysis are briefly outlined.

## 2. DNA-Assisted Amplification Strategies

In the past several years, DNA-assisted amplification technologies have received significant attention in biosensing because of their unique structure and properties. Benefiting from the advantages of specific Watson–Crick base pairing and their highly flexible design, DNA molecules can be self-assembled into various DNA structures, such as into DNA dumbbell structures [19], DNA flowers [20], and DNA tetrahedrons [21]. Additionally, signal amplification can be achieved by the governing of the DNA circuits through target triggering using the DNA’s programmable operation ability [22]. For example, ECL signal enhancement can be achieved through the target trigger 3D DNA walker moving continuously and automatically along the designed tracks [23,24]. In short, DNA amplification strategies can be classified into two categories: enzyme-assisted amplification and enzyme-free amplification strategies. The former involves enzymes and includes classical polymerase chain reaction (PCR), rolling circle amplification (RCA) or hyperbranched RCA (HRCA), endonuclease- and exonuclease-assisted amplification, and DNAzyme-involved amplification, while hybridization chain reaction (HCR) and DNA walker-based amplification without enzymes are examples of nonenzymatic amplification strategies [25]. Combining these versatile amplification strategies with biosensors can enable remarkable signal enhancements. Several examples that have been reported in recent years are summarized in Table 1, and the details of the signal amplification strategies are discussed below.

**Table 1:** Examples of representative biosensors based on DNA signal amplification strategies that have been developed in recent years.

Targets	Signal Amplification Strategy	Detection Range	Limit of Detection	Ref.
miRNA	bHCR	0.05–500 fM	0.18 fM	[26]
pyrophosphatase	Cu^+^-catalyzed azide–alkyne cycloaddition (CuAAC) with high-efficiency hybridization chain reaction (HCR)	0.025–50 mU	8 μU	[27]
Bisphenol A	Ru(phen)_3_^2+^ can integrate into the grooves of HCR products (dsDNA)	2.0–50 × 10^3^ pM	1.5 pM	[28]
cTnI	Au nanoclusters and HCR signal amplification	5–5 × 10^4^ fg/mL	1.01 fg/mL	[29]
Human immunodeficiency virus DNA	Target DNA triggered RCA signal amplification (RCA)	100–1 × 10^8^ aM	27.0 aM	[30]
HPV DNA	Bovine serum albumin carrier platforms and hyperbranched rolling circle amplification	10–1.5 × 10^4^ fM	7.6 fM	[31]
Hg^2+^	Exonuclease III-assisted CRISPR/Cas12a	0–1 × 10^6^ fM	0.45 fM	[32]
BT63DNA	ExoIII enzyme-assisted hybridization chain reaction combined with nanoparticle-loaded multiple probes	0.1–1 × 10^4^ fM	0.036 fM	[33]
MicroRNA	A synergistic promotion strategy for 3D DNA walker amplification	10–1 × 10^8^ aM	2.9 aM	[34]
miRNA-141	3D DNA walker-assisted CRISPR/Cas12a trans-cleavage	1–1 × 10^7^ fM	0.33 fM	[35]
SARS-CoV-2	Target DNA-participated entropy-driven amplified reaction	1–1 × 10^5^ fM	2.67 fM	[36]
MicroRNA let-7a	Swing arm location-controllable DNA walker based on the DNA tetrahedral nanostructures (DTNs)	10–1 × 10^8^ fM	4.92 fM	[37]
8-hydroxy-2′-deoxyguanosine	Target-induced multi-DNA release and nicking enzyme amplification strategy	100–1 × 10^7^ fM	25 fM	[38]
ochratoxin A	Nicking endonuclease-powered DNA walking machine	0.05–5 nM	0.012 nM	[39]
Myocardial miRNA	DNAzyme-regulated resonance energy transfer	10–1 × 10^7^ fM	2.44 fM	[40]
carcinoembryonic antigen	DNAzyme-driven DNA walker amplification	1–1 × 10^8^ fg/mL	0.21 fg/mL	[41]
5-Hydroxymethylcytosine	DNAzyme motor triggered by strand displacement amplification	1–1 × 10^6^ fM	0.49 fM	[42]
MircoRNA-21	Localized DNA cascade reaction (LDCR) in a DNA nanomachine	100–1 × 10^9^ aM	10.7 aM	[43]

### 2.1. Enzyme-Assisted DNA Amplification Strategies

**Figure 1:**
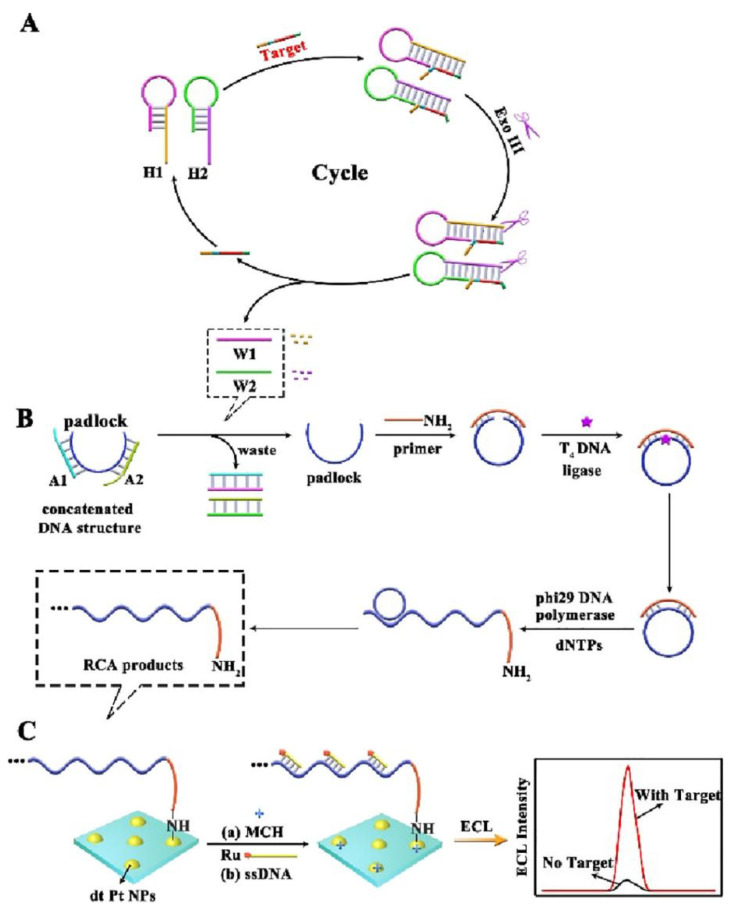
Example of the sensitive biosensor explored for HIV DNA fragment detection: (**A**) double recognition-triggered single-target recycling and (**B**) concatenated DNA structure-controlled rolling circle amplification; (**C**) construction process of the proposed ECL. Reproduced with permission from [30]. Copyright 2021, American Chemical Society.

As they are a type of enzyme, polymerases can catalyze DNA and RNA synthesis. They can replicate DNA and form long, linear, tandem, or repetitive chains of DNA with the assistance of a polymerase enzyme from the DNA template, primers, and deoxy-ribonucleoside triphosphate (dNTP) [44]. Polymerase chain reaction (PCR) remains the traditional and the “gold standard” enzyme-assisted DNA amplification strategy in bioanalysis due to its high sensitivity and low cost [45]. However, it has significant disadvantages, including the requirement of sophisticated and complicated processes and the presence of false-positive signals, which limit its practical use in the ECL domain. As alternative polymerase-based amplification techniques, rolling circle amplification (RCA) and hyperbranched RCA (HRCA) have attracted more attention, as they not only inherit isothermal amplification, but also promote improving the amplification efficiency. RCA requires a circular probe and DNA or RNA primers. In the presence of polymerases, prolonged extended ssDNA or double-stranded DNA is synthesized from the primer and the circular probe, and the ECL signal is enhanced based on the RCA product that it is loaded with or based on the in situ form abundant in the ECL luminophores. For example, as shown in Figure 1, two hairpin DNAs (H1 and H2) hybridize with the target DNA, releasing two output DNAs (W1 and W2) with the aid of exonuclease III. Subsequently, the concatenated DNA structure can be opened and can unlock the padlock oligonucleotide probe after being hybridized with two output DNAs. Under the action of the T4 DNA ligase, padlock DNA and other DNA primers are ligated to initiate the RCA reaction. Afterward, a prolong-extended ssDNA complementary to ruthenium (Ru)-labeled ssDNA is produced. Therefore, massive ruthenium (Ru)-labeled ssDNA is captured by the RCA products to generate a remarkable ECL signal, resulting in a large increase in amplification efficiency. The biosensor shows the highly specific and ultrasensitive detection of human immunodeficiency virus (HIV) DNA fragments when the detection limit is down to 27.0 aM [30]. Yen et al. utilized the RCA strategy to produce massive, long ssDNAs with a pH-dependent i-motif forming sequence, which was able to bind with hemin and catalyze the ECL reaction with the assistance of luminol/H_2_O_2_ solution. Because the i-motif structure is sensitive to pH change, a novel solid-state sensor for pH detection with a wide dynamic range from pH 4.0 to 7.4 was proposed [46]. To further improve the reaction efficiency, He et al. fabricated a high-reproducibility-and-sensitivity ECL biosensor for human papillomavirus 16 E6 and E7 by employing bovine serum albumin as a carrier platform to improve local steric hindrance and the HRCA strategy. After the addition of the target HPV DNA, HRCA occurred. Abundant HRCA products with double-stranded DNA (dsDNA) fragments of different lengths were generated, providing enough double helix space for the insertion of dichlorotris (1,10-phenanthroline) ruthenium(II) hydrate [Ru(phen)_3_]^2+^, which was acting as an ECL indicator, and releasing a strong and easily detected ECL signal [31]. Most RCA or HRCA-based ECL biosensors have shown great potential to avoid false-positive signals while also improving the sensitivity.

Another commonly used enzymatic amplification technology in the ECL domain is cleaving enzyme-assisted amplification, which can be divided into two types according to the function of the enzyme used: (1) endonucleases, including the nicking endonuclease (NEase) and duplex-specific nuclease and (2) exonucleases, including exonuclease I, exonuclease III, and T7 Exo. Cleaving enzymes are a class of enzymes that preferentially cleave the phosphodiester bonds of nucleic acids. The released DNA is recycled in the next round, leading to multiple cycles of signal amplification induced by multiple capture and release cycles of the target [39,47]. Zhao et al. fabricated an aptasensor for 8-hydroxy-2′-deoxyguanosine detection for early diagnosis via a target-induced multi-DNA release and nicking enzyme amplification strategy. Aptamers on magnetic beads were hybridized with three kinds of short DNA. After the aptamer specifically recognized the target, the three kinds of short DNA were released, and three-fold signal amplification increases were obtained. Following that, the Fc-labeled DNA hybridized with the released DNA with the help of a nicking endonuclease (Nt.AlwI). The substrate strand (Fc-HP) was cleaved into two parts, and the Fc-labeled DNA could then leave the surface of the electrode, resulting in a strong ECL intensity. At the same time, the three kinds of short DNA were released again and were reused to initiate the repeated hybridization–cleavage cycles because of the nicking endonuclease-assisted recycling amplification [38].

DNAzyme is a functional DNA molecule that shows catalytic activity similar to that of traditional protein enzymes. DNAzyme normally contains a substrate strand containing an embedded cleavage site and a binding site for metal ions (e.g., Pb^2+^, Cu^2+^, Mn^2+^, and Zn^2+^). The binding of metal ions triggers the catalytic activity of DNAzyme and the subsequent splitting of the substrate strand [40,48,49,50,51,52]. Therefore, as a metal ion-dependent enzyme, DNAzyme has been incorporated into sensors for the detection of various metal ions. Currently, DNAzymes are usually designed as a trigger to release the initiator DNA sequence, with the potential to further initiate other amplification reactions [53]. Therefore, DNAzymes are often integrated with other DNA amplification strategies to develop multiple-signal amplification-based biosensors, which can further improve ECL performance. By combining DNAzyme with cascading amplification, Sun et al. [54] developed an ultrasensitive and multi-targeted ECL sensing platform for the analysis of myocardial miRNAs. Three myocardial miRNAs were successfully detected to have a detection limit as low as 29.6 aM.

Despite the participation of enzymes greatly improving the sensitivity of biosensors, enzymes require strict experimental conditions to maintain the catalytic activity, with examples of conditions including pH and temperature. Furthermore, the involvement of enzymes increases the experimental costs and the number of complex procedures.

### 2.2. Enzyme-Free Amplification Strategies

**Figure 2:**
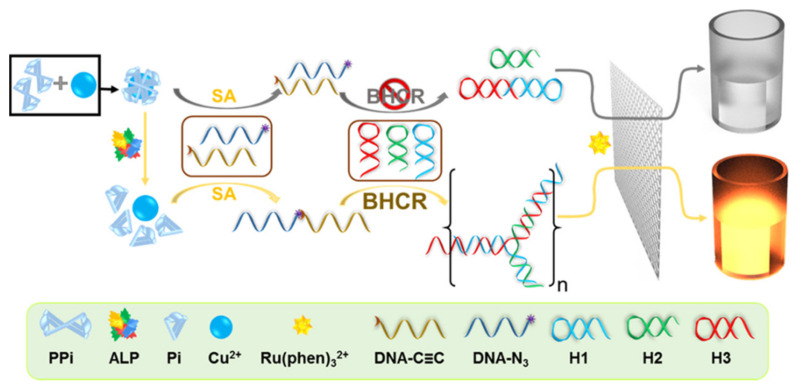
Example of the ECL biosensor for ALP detection based on BHCR amplification. Reproduced with permission from [60]. Copyright 2021, American Chemical Society.

Various nonenzymic DNA amplification technologies, such as hybridization chain reaction, catalyzed hairpin assembly [55], and entropy-driven catalysis [36], have been used for the fabrication of enzyme-free biosensing platforms. Among these techniques, hybridization chain reaction (HCR) has been widely integrated into ECL biosensor development to construct nonenzymatic DNA biosensors [56]. Many reviews focused on traditional linear HCR assembly, and novel forms of HCR have been discussed [57]. Traditional HCR assembly requires single-strand initiator DNA and two harpin fuel DNAs. Single-strand initiator DNA hybridizes with the two fuel DNAs to form a long linear double-stranded DNA polymer under mild conditions, enabling more ECL reagents such as [Ru(phen)_3_]^2+^ to be embedded into the dsDNA or more ECL reagent-labeled DNA to be bound to the DNA, resulting in remarkable ECL improvement [58,59]. However, HCR’s reaction efficiency is low due to its restricted presence on the electrode surface, which limits the efficiency of signal amplification. Lin’s group proposed a sensitive electrochemiluminescence biosensor based on a click chemistry-triggered hybridization chain reaction for pyrophosphatase detection in a homogeneous solution. The hybridization chain reaction was processed in a homogeneous solution, which obviously improved the amplification efficiency [27]. With the development of molecular programming, diverse nucleic acid reaction circuits based on HCR have been proposed to form branched or dendritic nanostructures. Lin’s group successfully integrated branched hybridization chain reaction (BHCR) into a biosensing approach (Figure 2). BHCR, which is a derivative of HCR, possesses multiple unique reaction orientations that greatly accelerate the reaction and improve the amplification efficiency. Therefore, BHCR not only inherits the properties of the nonenzymatic and isothermal amplification characteristics of HCR. However, it also possesses the advantages of rapid reaction kinetics and a high amplification efficiency because of its unique multiple reaction orientations [60].

**Figure 3:**
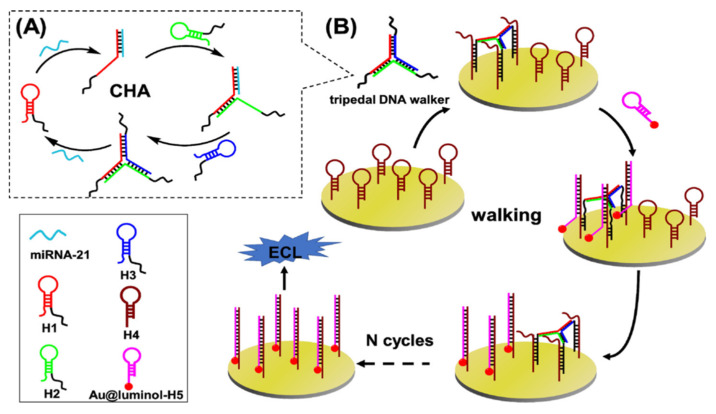
Example of the ECL biosensor for miRNA-21 detection based on the DNA walker strategy: (**A**) generation process of the tripedal DNA walker; (**B**) walking cycles of the DNA walker. Reproduced with permission from [22]. Copyright 2020, American Chemical Society.

Additionally, benefiting from DNA molecular programming, nanomachine-based amplification strategies have started to receive attention. Various DNA structures with different functional properties, such as DNA motors, DNA robots, and DNA walkers, have been designed and exploited for the construction of high-sensitivity biosensors. Especially, DNA nanomachines can be integrated for the quantitative detection of biomolecules using different amplification strategies [61]. As an example, DNA walkers, which are a type of autonomous nanomachine, show broad special cascade signal enhancement characteristics that are generated by their autonomous movement along the designed track. To date, most DNA walkers based on one walking strand are referred to as a single-leg DNA walkers; therefore, the walking area is limited, and the immediate response is slow. To overcome the above issue, some efforts have been devoted to the development of ECL strategies using multipedal DNA walkers by attaching multiple walking strands. It is worth mentioning that DNA walkers normally need to be driven by other strategies, such as DNAzyme, endonuclease-mediated hydrolysis, or toehold-mediated strand displacement (TMDR). As shown in Figure 3, Wang et al. adopted catalytic hairpin assembly (CHA) to trigger a tripedal DNA walker in the presence of miRNA-21. The tripedal DNA walker formed a Y-shaped structure with three “legs” and walked on a DNA track, producing a significant ECL signal. Combining CHA and tripedal DNA walker to develop a dual signal amplification strategy, high-sensitivity miRNA-21 detection was achieved, with a superior detection limit of 4 aM and a broad linear range of 10 aM to 1 pM [22].

## 3. Efficient ECL Luminophores

For now, most of the reported ECL biosensors and all commercial ECL assays are developed based on the ECL luminescent reagent tris(2,2′-bipyridineruthenium(II) ([Ru(bpy)_3_]^2+^) [22,62]; however, the quantum yield of [Ru(bpy)_3_]^2+^ is low, which can limit the sensitivity of the assays. Cyclometalated iridium complexes, which exhibit higher luminescence efficiency and long excited state lifetimes, have been reported as alternative luminescent reagents for [Ru(bpy)_3_]^2+^. The ECL efficiency from (pq)_2_Ir(acac) is reported to be significantly higher than that of [Ru(bpy)_3_]^2+^ [63]. Cyclometalated iridium complexes also allow the ECL emission to be tuned from the visible region to the UV region, which promotes the appearance of resolvable “mixed-ECL” from solutions containing multiple luminophores [64]. However, the poor water solubility of iridium complexes limits its practical use.

**Figure 4:**
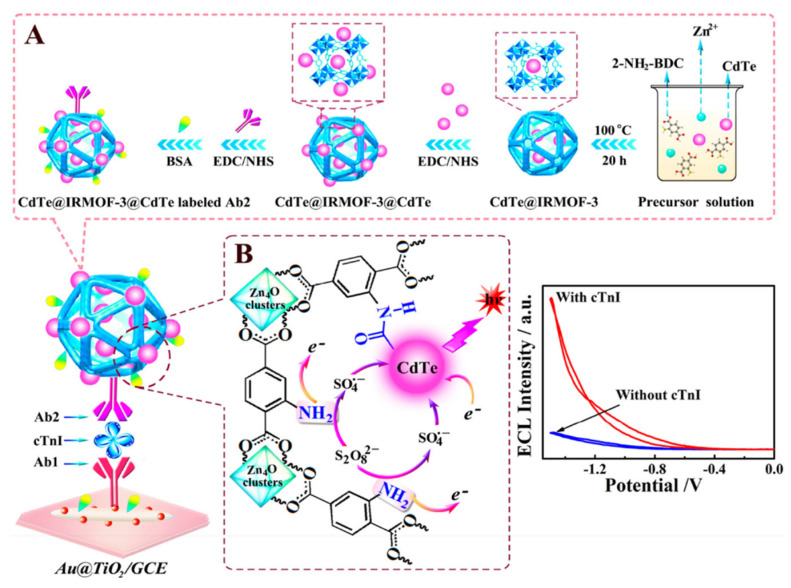
(**A**) Preparation of the signal probe. (**B**) Possible mechanism of IRMOF−3 accelerator-mediated enhancement of cTnI detection in the CdTe/S_2_O_8_^2−^ system. Reproduced with permission from [70]. Copyright 2018, American Chemical Society.

In the past few decades, experimental and theoretical studies have shown that quantum dots possess unique ECL properties; however, the ECL intensity of QD is unstable and weak [65]. To meet the demands for efficient luminophores, nanocarriers loaded with QDs demonstrate excellent ECL performance, with signals being obviously amplified. In general, silica nanoparticles, carbon nanomaterials, metal organic frameworks (MOFs), and transition metal oxides are applied as nanocarriers to load QDs. To date, QD-based biosensors have been widely used for ultrasensitive analysis [66,67,68,69], which benefits from their unique features such as their excellent biocompatibility, water solubility, and low toxicity. Zhuo’s group [70] prepared IRMOF−3 accelerator-enriched QDs (CdTe@IRMOF−3@CdTe) using a direct encapsulation method for the trace detection of cTnI. With S_2_O_8_^2−^ as the coreactant, the composites showed enhanced ECL intensity compared to other QD aggregates. Moreover, IRMOF−3 can be functioned as a coreactant accelerator that can further amplify the ECL signal. The developed sensor exhibited a wide dynamic range of 1.1 fg/mL to 11 ng/mL for cTnI, with a limit of detection (LOD) of 0.46 fg/mL (Figure 4).

Recently, certain metal nanoclusters have also been found to possess ECL properties [71]. To date, gold nanoclusters have emerged as a new class of ECL emitters due to their stable optical and electrochemical properties, monodispersity size, and low- and well-defined band gap and high atomic accuracy [72]. Nie et al. [73] reported a novel luminophore, a Au NC-based metal-organic framework (Au NC-based MOF) that showed 10-fold-enhanced anodic ECL efficiency over aggregated GSH-Au NCs in an aqueous solution, and when rutin was the model analyte, a low detection limit of 10 nM was achieved.

Other types of nanomaterials have recently been used in ECL bioanalysis, such as graphite-phase carbon nitride (CN) [74], a novel nitrogen-rich two-dimensional carbon material, which has a wide range of applications in photocatalysis and biosensing [75,76]. Surface-modified CN is of great significance due to its practical application, especially for the regulation of its ECL characteristics [77,78]. Ji et al. [79] conducted a systematic scientific investigation on the application of graphite-phase carbon nitride (CN) in the ECL field. They destroyed the stacking between layers of CN using a simple mechanical grinding method to obtain CN of a smaller size. The prepared CN nanocrystals not only retained the photoelectric characteristics of the original CN but were also able to overcome the CN defects on the surface of the materials, which provided a basis for its further application in the field of biosensors.

In total, various kinds of nanomaterials, including carbon-based nanomaterials, metal nanomaterials, and other inorganic or organic nanomaterials, as have been used as coreactant or luminophores to enhance ECL performance in biosensing. However, the reported mechanism proposed that the ECL of nanomaterials be generated through both the electron and holes when the dots are injected separately onto the surface. The surface states of nanomaterials are reflected in the ECL performance. The ECL response can be increased by increasing the efficiency of electron–hole recombination via different strategies.

The ECL is much more sensitive to the surface states of CDs. Thus, developing unique luminophores and especially nanomaterials that enhance ECL efficiency as well as enable sensitive targets sensing remains a challenging research area, and the development of advanced nanomaterials is an important research direction in the design of amplified ECL biosensors.

## 4. Ratiometric Strategies

### 4.1. Potential-Resolved Ratiometric Strategies

**Figure 5:**
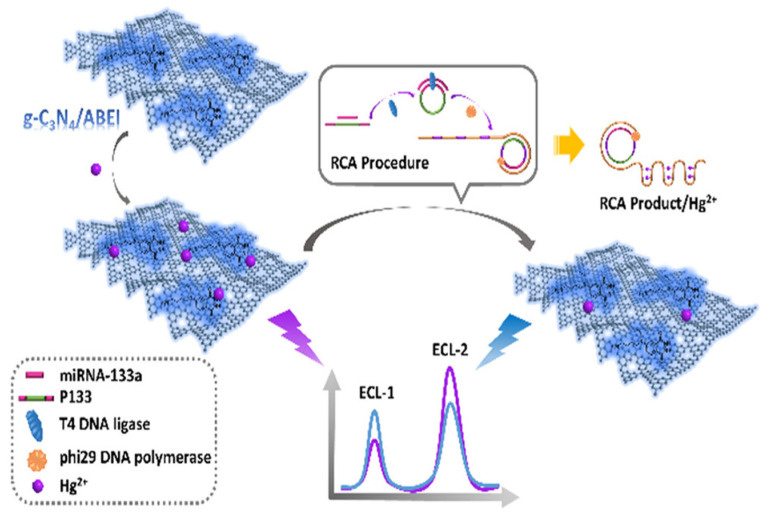
Potential-resolved ratiometric strategy for the detection of miRNA-155. Reproduced with permission from [25]. Copyright 2020, American Chemical Society.

Potential-resolved strategies require two ECL emitters that emit light at different potentials [80]. The difference in the peak emission potential between the two ECL peaks should be enough to be easily distinguished without interference [81]. Based on the intensity ratio of the two ECL peaks at different potentials, many ratiometric biosensors have been established for the detection of prostate-specific antigen (PSA), aflatoxin B1 (AFB1), dopamine, miRNA, metal cations, and cancer cells, which not only improve the sensitivity, but also avoid false-positive errors in complex matrices [24,82]. However, this kind of ratiometric biosensor also requires two coreactants in addition to additional luminophores [44,83,84], which increase the complexity of the whole experimental process. Some efforts have been made to develop single-luminophore-based ratiometric sensors [24]. As shown in Figure 5, Cui’s group fabricated a ratiometric biosensor for the determination of miR-133a. The novel ECL luminophores, graphitic carbon nitrides (g-C_3_N_4_) functionalized by N-(aminobutyl)-N-(ethylisoluminol) (ABEI) (g-C_3_N_4_/ABEI), were used as the sensing interface. g-C_3_N_4_ emitted ECL light in the negative potential range at −1.6, while the ECL emission of ABEI occurred in the positive potential range at +1.2 in the presence of H_2_O_2_. Based on the ECL intensity ratio of the g-C_3_N_4_/ABEI, the proposed bioassay presented a linear range from 0.1 fM to 1.0 pM and a LOD of 48.0 aM. Cao’s group fabricated a potential-resolved ECL biosensor based on novel nano-luminophores: nano-graphene oxide wrapped titanium dioxide (nGO@TiO_2_NLPs), which showed potential-resolved ECL properties in a neutral aqueous solution using K_2_S_2_O_8_ as a coreactant. These new luminophores could also be employed as luminescent cathode and anode materials simultaneously. In the presence of cardiac troponin I (cTnI), the aptamer protrudes from the electrode surface owing to its rigidity, leading to a reduction in the charge transfer resistance of the modified working electrode and a ratio enhancement of the two ECL signals of the nGO@TiO_2_ NLPs. According to the increased ECL ratio, cTnI was quantified using the ratiometric ECL aptasensor, with a linear dynamic range of 1.0 × 10^−13^–1.0 × 10^−10^ mol/L.

### 4.2. Spectrum-Resolved Ratiometric Strategies

**Figure 6:**
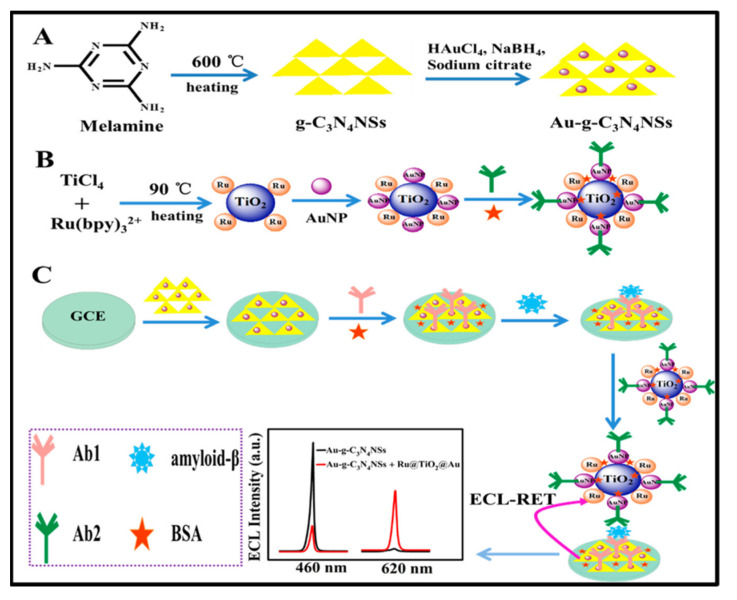
(**A**) Preparation of Au-g-C_3_N_4_NSs; (**B**) fabrication of Ru@TiO_2_@Au-Ab2 conjugate; and (**C**) assembly process of DWR-ECL immunosensor. Reproduced with permission from [88]. Copyright 2021, American Chemical Society.

Compared to potential-resolved sensors, spectrum-resolved ECL sensors not only inherit the advantages of high accuracy but can also be used in a narrow potential range [85,86]. These sensors require ECL emitters that emit light at different wavelengths [87], and there should also ideally be overlap between the ECL emission and absorption peaks to allow resonance energy transfer (RET) to occur. For example, based on ECL RET, a dual-wavelength ratiometric ECL sensor was established for the detection of the amyloid-β protein in human serum [88]. The process is displayed in Figure 6. A couple of emitters, Ru@TiO_2_@Au nanomaterial/gold nanoparticle (AuNP)-modified graphitic carbon nitride nanosheets (g-C_3_N_4_NSs), were employed as the energy receptor and energy donor. With the addition of a target protein, due to the RET effect between the two emitters, the g-C_3_N_4_NSs signal at about 460 nm was gradually quenched, while the signal of Ru@TiO_2_@Au increased at about 620 nm. During the experiment, based on the ratio of I_460nm_/I_620nm_, the linearity range of Aβ42 was found to be from 1 × 10^–5^ to 200 ng/mL, with a limit of detection (LOD) of 2.6 fg/mL, which makes the sensor suitable for application in clinical diagnosis.

## 5. Surface-Enhanced Strategies

**Figure 7:**
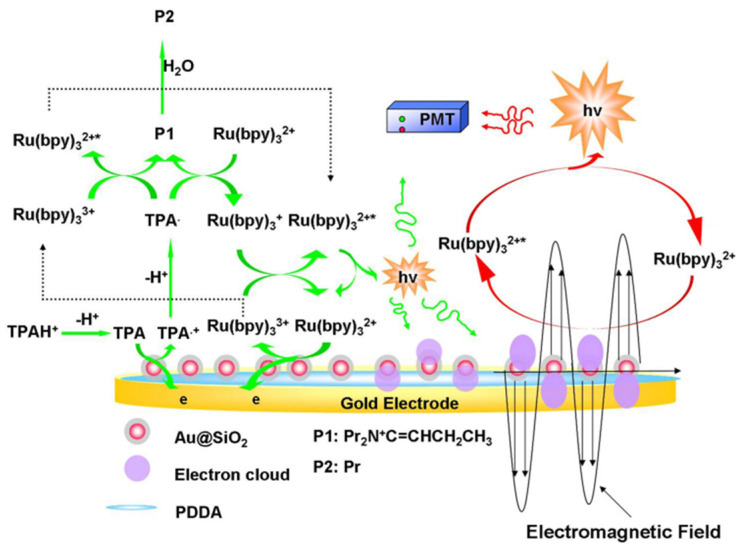
The proposed SEECL mechanism. Reproduced with permission from [94]. Copyright 2015, Scientific Reports.

Previous reports have demonstrated that the localized surface plasmon resonance (LSPR) of metal nanoparticles (such as gold and silver) can significantly enhance the spectral signal [89,90]. LSPR is a physical phenomenon that is generated when the surface plasma of noble metal nanoparticles is irradiated by incident light with the same frequency [91]. LSPR can generate a local electromagnetic field around noble metal nanoparticles, thus enhancing the spectral signal [92]. By controlling the distance between the surface of noble metal nanoparticles and the ECL luminophore, the intensity of ECL can be greatly improved [93]. This phenomenon is called surface-enhanced electrochemiluminescence (SEECL). As shown in Figure 7, credible evidence of this process as well as a detailed mechanism of it has been presented by Wang and co-workers [94]. The ECL signal of the Au NP@SiO_2_-modified electrode was 10 times higher than that of the bare electrode. Since this initial report on SEECL, a series of ultra-sensitive biosensors based on SEECL have been developed [90,93,95,96,97,98]. For example, an ultra-sensitive biosensor for Hg^2+^ using the local surface plasmon resonance (LSPR) of gold nanorods (Au NR) has been proposed. When Hg^2+^ is present, the conformation of ssDNA probes changed into a hairpin structure by forming a T-Hg^2+^-T structure. [Ru(bpy)_3_]^2+^ can be embedded into a hairpin DNA probe to generate ECL emissions, while the LSPR of Au NRs can enhance ECL emissions. As the Hg^2+^ concentration increases, the ECL intensity also increases, and the detection limit of the sensor reaches 10 fM [99]. The LSPR of metal nanoparticles is also often used to enhance the electrochemiluminescence of quantum dots. CuZnInS quantum dots are a novel ECL luminescent material, but they suffer from a low ECL efficiency. Based on CuZnInS quantum dots (QDs) and gold nanoparticles (AuNPs), Chen et al. [97] developed a novel DNA electrochemiluminescence sensor for the highly sensitive detection of the epidermal growth factor receptor (EGFR) gene closely related to lung cancer. The detection range of EGFR ranged from 0.05 to 1 nmol/L, and the detection limit was 0.0043 nmol/L. Apart from Au monomers, Au–Au dimers can be applied in the construction of biosensors to further improve the signal intensity and the sensitivity due to the surface plasmon coupling between two Au monomers, resulting in high electromagnetic field enhancement [100]. It is worth noting that the metallic substrates used in most surface-enhanced strategies are limited to Au nanomaterials. Although Au nanomaterials are easily synthesized and have good stability, other nanomaterial candidates should be explored for the purposes of determining additional excellent plasmonic properties. Therefore, it is worth noting that Ag nanomaterials have also shown excellent plasmonic properties. Cao et al. employed AgNP nanocrystals as the LSPR source and MoS_2_ QDs as the ECL emitter, which demonstrated that Ag nanomaterials can greatly enhance the effects on ECL. The detection limit for microRNA-21 was 0.2 fM. 

In addition, imaging technology coupled with surface-enhanced electrochemiluminescence has been used in biosensing [14,101]. Liu and his team [102] constructed a biosensor for the determination of *Escherichia coli* through the mode conversion between resonance energy transfer (ECL-RET) and surface plasmon-coupled ECL (SPC-ECL) by controlling the distance between the BN QDs and Au NPs. The BN QDs and Au NPs were separately modified at the two ends of the hairpin DNA. When there was no target, resonance energy transfer occurred due to the close distance between the BN QDs and Au NPs, and the ECL signal of the BN QDs was inhibited by the AuNPs. In the presence of the target, the hairpin DNA hybridized with the target, and the structure changed to a linear conformation; therefore, as the distance between the BN QDs and Au NPs increased, the ECL intensity also increased. As a result, ECL-RET is replaced by the SPC-ECL effect, and the ECL signal is enhanced. In addition, the author presents the ECL signal for the first time using a CMOS camera imaging method and a smartphone. The fabricated biosensor showed a LOD of 0.3 pmol/L, with a linear range from 1 pmol/L to 5 nmol/L.

Compared to traditional biosensors, biosensors based on surface-enhanced strategies are simpler and more sensitive and show great potential for the detection of ultra-trace biomarkers for clinic diagnostics. Moreover, biosensors can be more biocompatible owing to the choice of Au or Ag nanomaterials as an LSPR source. However, real sample analysis is still a significant challenge.

## 6. Other Types of Signal-Amplification Strategies

Most biosensors mainly depend on single-signal output, which make them easily susceptible to other intrinsic and extrinsic factors. Single-signal assays in particular cannot meet the increasing demands for clinical diagnosis because some diseases are related to one or more biomarkers. Therefore, many efforts have been devoted to developing ECL biosensors based on multiple-signal outputs, which enable multiple analytes to be measured simultaneously. For example, Su’s group [64] successfully synthesized three novel ECL emitters, including ruthenium and iridium complexes. Based on the ECL emitters with different spectral peaks at different potentials, a multiplex immunoassay for the simultaneous detection of carcinoembryonic antigen (CEA), alpha-fetoprotein (AFP), and beta-human chorionic gonadotropin (β-HCG) was developed. However, it is challenging to develop novel luminophores with potential-resolved or spectrum-resolved properties because the number of luminophores pairs for multiplex immunoassays is limited.

It is well-known that the coreactant process is one of the main mechanisms of ECL. Thus, the coreactant plays an important role during signal transduction, and the development of novel and efficient coreactants is another amplification strategy for biosensor construction. Nanomaterials, which are emerging as a new class of coreactants, have been exploited as attractive candidates for inorganic molecules, especially carbon dot-based nanomaterials. For instance, Wang’ group [103] proved that the use of boron nitride quantum dots as a coreactant to enhance the ECL of [Ru(bpy)_3_]^2+^ resulted in a 400-fold enhancement being achieved. This is due to amino-bearing groups on the surface of the quantum dots. Thus, enhanced ECL efficiency is highly related to the surface state of nanomaterials [104].

## 7. Conclusions and Perspectives

Signal-amplification-based biosensors have gained great attention and have undergone rapid development owing to the requirements for ultrasensitive biosensors and trends towards early clinical diagnosis. Signal amplification strategies open novel approaches for developing ultrasensitive bioassays with a wide dynamic range. Specifically, they offer opportunities for monitoring early diagnosis, monitoring disease progression, and predicting disease in biomedical diagnoses. Among these strategies, DNA-assisted techniques are the most popular for signal amplification during ECL bioassays due to the advantages of specific base pairing, programmable operation, and predictable assembly. Enzyme-assisted DNA amplification strategies have achieved enhanced sensitivity in ECL, but enzymatic reactions are susceptible to environmental factors, which ultimately affect the DNA amplification efficiency and limit their application in complex biological systems. Therefore, the development of low-cost, sensitive, and enzyme-free strategies is the research direction to achieve future commercialization. However, the commercialization of point of care testing is still in its early stages. Multiple DNA circuits make the processes more complicated, and the amplification efficiency at each step is still unknown, affecting the detection accuracy. Therefore, highly efficient luminophores have been explored to amplify ECL signals. Finally, ratiometric strategies and surface-enhanced ECL approaches have been introduced into biosensing, which can not only improve the sensitivity, but can also increase the accuracy. This review presents the recent progress in ECL biosensors that have been integrated with various kinds of signal amplification strategies, hoping to provide guidance for designing novel ECL biosensors.

Based on the above-mentioned strategies, ECL biosensors have the ability to achieve the ultra-trace level detection of targets. However, most biosensors are only demonstrated to be successful in their principle of concept and the translation of research into industrial manufacturing and marketing remains a significant challenge. To achieve commercialization, there is urgency to develop disposable, low-cost, and lab-on-chip ECL platforms to conduct tests with a rapid response time and that have high convenience. Specifically, ECL systems that are autonomous and miniaturized and that show great potential in practical applications represent future research trends. In addition, the development of various low-cost photodetectors such as CCD cameras, smartphone cameras, and other imaging techniques coupled with ECL biosensors can realize multi-component analysis and single-molecule detection. Single-biomolecule ECL imaging should be the subject of major efforts to greatly expand ECL applications in bioanalysis.

## Data Availability

Not applicable.
